# Loss of succinyl-CoA synthase ADP-forming β subunit disrupts mtDNA stability and mitochondrial dynamics in neurons

**DOI:** 10.1038/s41598-017-05168-5

**Published:** 2017-08-02

**Authors:** Yujun Zhao, Jing Tian, Shaomei Sui, Xiaodong Yuan, Hao Chen, Chuanqiang Qu, Yifeng Du, Lan Guo, Heng Du

**Affiliations:** 1 Shandong University, Shandong Qianfoshan Hospital, Jinan, Shandong Sheng China; 2 Yantai Yuhuangding Hospital, Yantai, Shandong Sheng China; 3 The University of Texas, Dallas, Richardson USA; 4 Jinan Central Hospital, Jinan, Shandong Sheng China; 5 Shandong University, Shandong Provincial Hospital, Jinan, Shandong Sheng China

## Abstract

Succinyl Coenzyme A synthetase (SCS) is a key mitochondrial enzyme. Defected SCS ADP-forming β subunit (SCS A-β) is linked to lethal infantile Leigh or leigh-like syndrome. However, the impacts of SCS A-β deficiency on mitochondria specifically in neurons have not yet been comprehensively investigated. Here, by down-regulating the expression levels of SCS A-β in cultured mouse neurons, we have found that SCS A-β deficiency induces severe mitochondrial dysfunction including lowered oxidative phosphorylation (OXPHOS) efficiency, increased mitochondrial superoxide production, and mtDNA depletion as well as aberrations of mitochondrial fusion and fission proteins, which eventually leads to neuronal stress. Our data also suggest that the deregulation of mitochondrial nucleoside diphosphate kinase (NDPK) together with defects in mitochondrial transcription factors including mitochondrial DNA pol γ and Twinkle contribute to SCS A-β deficiency-mediated mtDNA instability. Furthermore, we have found that SCS A-β deficiency has detrimental influence on neuronal mitochondrial dynamics. Put together, the results have furnished our knowledge on the pathogenesis of SCS A-β deficiency-related mitochondrial diseases and revealed the vital role of SCS A-β in maintaining neuronal mitochondrial quality control and neuronal physiology.

## Introduction

Succinyl Coenzyme A synthetase (SCS) is a key mitochondrial enzyme involved in tricarboxylic acid (TCA) cycle^1^. Structurally, mammalian SCS is in a form of αβ heterodimer, which is composed of two entities including one invariant α subunit (SCS-α) encoded by *SUCLG1* and a variant β subunit. SCS β subunit has two isoforms known as the ADP-forming β subunit (SCS A-β) encoded by *SUCLA2* and the GDP-forming β subunit (SCS G-β) encoded by *SUCLG2* ^2^. The incorporation of SCS A-β or G-β with SCS-α catalyzes an ADP- or GDP-dependent reaction, respectively^2^.

Although little is known about the mechanisms regulating the expression of the two SCS β isoforms in different cell types, previous studies have shown a significantly higher abundance of SCS A-β over G-β in brain tissues^2–4^. Such finding seems to suggest that SCS A-β is critical for neuronal physiology. This has been supported by further clinical observations that mutations in *SUCLA2* cause loss of SCS A-β function resulting in lethal neurological disorders including infantile Leigh or Leigh-like syndrome^5–7^. Furthermore, *SUCLA2* +/− mice demonstrated pronounced brain mitochondrial dysfunction^8^. In addition to defected mitochondrial bioenergetics, mtDNA depletion is a featured pathology in patients carrying *SUCLA2* mutants^7, 9, 10^. Previous studies have attributed such mtDNA depletion to the deactivation of mitochondrial nucleoside diphosphate kinase (NDPK), which leads to dampened mitochondrial deoxyribonucleotide (dNTP) metabolism^9^. However, whether SCS A-β deficiency also affects mitochondrial replication factors has never been comprehensively studied in neural cells.

Mitochondria are highly dynamic organelles that constantly change their morphology through fusion and fission to accommodate the energy demand of their host cells. Mitochondrial dynamics in normal fashion is extremely important for the survival and activity of neurons^11, 12^; and abnormal mitochondrial dynamics are closely associated with neuronal injury^13–15^. Therefore, it is generally believed that mitochondrial dynamics and mitochondrial function are reciprocally affected^16, 17^. In this regard, it would be of great interest to determine whether SCS A-β loss-of-function impacts neuronal mitochondrial dynamics. However, to date there is no clear answer to this question.

To address these questions, we down-regulated SCS A-β expression in primary cultured mouse neurons. We have found that loss of SCS A-β suppresses SCS-α expression and induces severe mitochondrial dysfunction and synaptic loss. SCS A-β deficiency-induced mtDNA instability is likely to be the convergence of reduced expression levels of NDPK and Twinkle as well as mitochondrial DNA polymerase γ (DNA pol γ). Lastly, deregulated mitochondrial dynamics is a pronounced detrimental influence of SCS A-β deficiency on neurons.

## Experimental Procedures

### Primary neuron culture

Animal studies were approved and performed under the guidelines of Institutional Animal Care and Use Committee (IACUC) at Shandong University, and the University of Texas at Dallas as well as National Institute of Health. C57BL/6 J mice were purchased from Jackson Lab. Neurons were cultured as previously described^18^. Briefly, cerebral cortex was dissected from Day 0 pups and kept in cold Hank’s balanced salt solution (HBSS, Sigma-Aldrich). Tissues were digested in 0.05% trypsin (Sigma-Aldrich) at 37 °C for 15 min to dissociate neurons. Mouse primary neurons were cultured in neuron culture medium (Neurobasal A with 2% B27 supplement, 0.5 mM L-glutamine, 50 U/ml penicillin, and 50 μg/ml streptomycin) with an appropriate density.

### SCS A-β knock-down in mouse primary neurons

Lentivirus expressing shRNA targeted to mouse *SUCLA2* were packaged with lentivirus shRNA construct (Sigma-Aldrich, Clone: SHCLNG-NM_011506), packaging vector psPAX2 (Addgene) and envelope vector pMD2.G (Addgene). Lentivirus expressing non-target shRNA control (Sigma-Aldrich, SHC202) were used as a control. The virus were harvested and concentrated through ultracentrifuge. The lentivirus titer was measured by Lenti-X qRT-PCR titration kit (Clontech). Neurons after 10 days in culture were employed for shRNA delivery by the infection with lentivirus at an m.o.i. of 5. The virus containing medium was removed after 2 hours followed by the replacement with fresh culture medium to continue the culture for 6 days before experiments.

### Immunoblotting analysis

Neurons were collected in 1X sample loading buffer (50 mM Tris-HCl pH 6.8, 2% SDS, 10% glycerol, 1% b-mercaptoethanol, 12.5 mM EDTA and 0.02% bromophenol blue) followed by a 10-min’s boiling. Proteins were separated by SDS–PAGE (Thermo Fisher Scientific, 12% Bis-Tris gel), and then transferred to PVDF membranes for blotting (Bio-Rad Laboratories). After blocking in TBS buffer (20 mM Tris-HCl, 150 mM sodium chloride) containing 5% (wt/vol) nonfat dry milk (Santa Cruz Biotechnology) for 1hr at room temperature, the membrane was incubated with primary antibodies overnight at 4 °C. After the incubation with the corresponding secondary antibody for 1 hr at room temperature, the blots were developed in enhanced chemiluminescence (ECL, Biorad). Images were collected on Bio-Rad Chemidoc Imaging System. Image J software (National Institutes of Health) was used for data analysis. The following antibodies were used: Rabbit antibody against *SUCLA2* (Genetex, GTX109728, 1:20000); Rabbit antibody against DNA pol γ (Santa Cruz Biotechnology, sc-48815, 1:1000); Rabbit antibody against MFN2 (Cell Signaling Technology, #9482, 1:1000); Mouse antibody against OPA1 (BD, 612606, 1:1000); Rabbit antibody against SSBP1 (Santa Cruz Biotechnology, sc-67101, 1:1000); Rabbit antibody against TOM40 (Santa Cruz Biotechnology, sc-11414, 1:2000); Rabbit antibody against Twinkle (Santa Cruz Biotechnology, sc-134915, 1:1000); Rabbit antibody against mitochondrial nucleoside diphosphate kinase (Cell Signaling Technology, #3345, 1:1000); Rabbit antibody against phospho-Drp1(ser616) (Cell Signaling Technology, #3455, 1:1000); Mouse antibody against Drp1 (BD, 611112, 1:1000); Rabbit antibody against Succinyl-CoA Synthetase (Cell Signaling Technology, #8071, 1:2000); Rabbit antibody against FIS1 (Thermo Scientific, PA1-41082, 1:500); Goat anti-mouse IgG HRP conjugated and goat anti-rabbit IgG HRP conjugated (Thermo Fisher Scientific, #626520 and 656120).

### Mitochondrial membrane potential assay and dendritic mitochondrial content measurement

Mitochondrial membrane potential was measured using Tetramethylrhodamine, methyl ester (TMRM) as we previously described^19^. MitoTracker Green FM (Thermo Fisher Scientific) was used to label mitochondria in live neurons. MitoTracker Green is a fluorescent dye targeted to the mitochondrial matrix regardless of the mitochondrial membrane potential and covalently bind to mitochondrial proteins by reacting with free thiol groups of cysteine residues. Neurons were co-incubated with 200 nM TMRM (Sigma-Aldrich) and 400 nM MitoTracker Green in neuron culture medium for 30 min in an incubator (5% CO_2_, 37 °C). After a wash with pre-warmed neuron culture medium, neurons were kept in the incubator for 15 min before image capture. The images were collected on a Nikon inverted microscope with on-stage incubator (5% CO_2_, 37 °C). The intensity of TMRM was subsequently analyzed by using Nikon NIS Advanced Research software. The dendritic mitochondrial content was calculated as the area of a dendrite occupied with Mitotracker Green-labeled mitochondria.

### Mitochondrial superoxide assay

Mitochondrial superoxide levels were determined by the staining of Mitosox Red (Thermo Fisher Scientific). Briefly, neurons were incubated with 1 μM MitoSox Red in neuron culture medium for 30 min in an incubator (5% CO_2_, 37 °C) followed by washing. Afterwards, neurons were fixed in 4% paraformaldehyde and subjected to DAPI staining to visualize nuclear. The images were collected on a Nikon confocal microscope. The intensity of Mitosox Red was subsequently analyzed by using Nikon NIS Advanced Research software.

### ATP content measurement

The ATP content level from the lysate of cultured neurons was analyzed by using ATP Luminescent assay kit (Abcam) following the manufacturer’s instructions.

### Mitochondria density, length and volume measurement

The cDNA encoding mitochondria targeted DsRed (from pDsRed-mito, Addgene) was inserted into lentivirus vector containing human polyubiquitin promoter-C (Addgene). Lentivirus were packaged with above lentivirus vector, psPAX2 and pMD2.G in HEK293T cells. Mouse primary hippocampal neurons were infected with lentivirus expressing DsRed-mito two days before the infection of lentivirus containing *SUCLA2* shRNA or nonTarget shRNA control. The neurons were fixed with 4% paraformaldehyde, then subjected to anti-MAP2 (microtubule associated protein 2 (MAP2)) (Sigma-Aldrich, M4403, 1:400), followed by goat anti-mouse IgG Alexa 488 (Invitrogen, A11029, 1:500). Images were collected on a Nikon confocal microscopy. Particles with strong labeling (in comparison with background) and clear edges confined in dendrites were considered as mitochondria. Mitochondria density, length and volume were measured and analyzed by using NIS image analysis software (Nikon). Mitochondrial volume was measured after 3-dimensional reconstruction of the images by using Nikon NIS Advance Research Software. Mitochondria located in dendritic segments between 30 and 60 nm from the soma were used for the analysis.

### Mitochondria DNA copy number measurement

The relative mtDNA copy numbers were quantified by real-time PCR and normalized by simultaneous quantification of nuclear DNA^20^. The genomic DNA was extracted by GeneJET genomic DNA purification kit (Thermo Scientific) according to the manufacturer’s protocol. Primers were designed to detect cytochrome b (forward 5′-gccaccttgacccgattcttcgc-3′ and reverse 5′-tgaacgattgctagggccgcg-3′) for mtDNA, and β-actin (forward 5′-ggactcctatgtgggtgacg-3′ and reverse 5′-aggtgtggtgccagatcttc-3′) for nuclear DNA. PCR reaction were performed using 20ng (mtDNA detection) or 0.5 ng (nuclear DNA detection) of total DNA, 8 nM of each primer and SYBR Premix Ex Taq (Clontech) in Mastercycler EP Realplex2 (Eppendorf). To minimize pipetting error, DNA was added to each well in 5 µl volumes. The amplification conditions were 95 °C for 5 min followed by 40 cycles of 95 °C for 15 s, 55 °C for 15 s and 72 °C for 20 s. The relative mtDNA copy number was calculated by the equation 2^−ΔCt^ (ΔCt = Ct mtDNA − Ct nuclear DNA).

### Synaptic density measurement

Synaptic density of cultured neurons was measured as we previously described^19^. Cultured neurons were fixed in 4% paraformaldehyde for 30 min followed by washing using PBS. Fixed neurons were blocked in 5% goat serum for 30 min at room temperature. Postsynaptic density protein 95 (PSD-95), vesicular glutamate transporter 1 (vGlut1) and MAP2 were labelled by rabbit anti-PSD95 (Cell Signaling, #3450, 1:200), guinea pig anti-vGlut1 (Synaptic System, #135304, 1:400) and mouse anti-MAP2 (Sigma-Aldrich, M4403, 1:400) followed by goat anti-rabbit IgG conjugated with Alexa 594 (Invitrogen, A11037, 1:500), goat anti-guinea pig IgG conjugated with Alexa 647 (Invitrogen, A21450, 1:500) and goat anti-mouse IgG conjugated with Alexa 488 (Invitrogen, A11029, 1:500), respectively. Images were captured by using a Nikon confocal microscope followed by three-dimensional reconstruction by using Nikon-Elements Advanced Research software. The synapses were defined by colocalization of vGlut1 and PSD95. Dendritic segments between 30 and 60 nm from the soma were used for the analysis.

### Statistical analysis

One-way ANOVA followed by Bonferroni post hoc analysis or Student t-tests wherever appropriate were used for repeated measure analysis on SPSS software (IBM software). The distribution and variance were normal and similar in all groups. P < 0.05 was considered significant. All data were expressed as the mean ± s.e.m.

## Results

### Loss of SCS A-β reduces SCS-α expression

**Figure 1:**
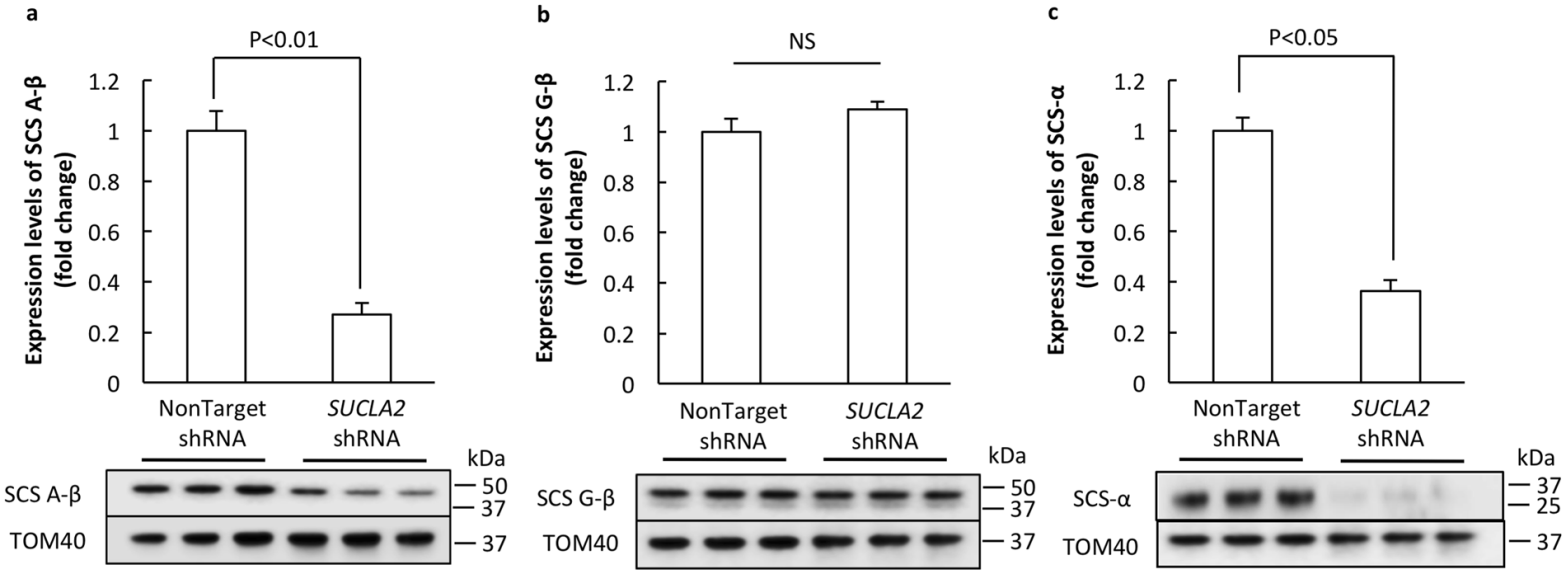
Down-regulation of SCS A-β inhibits SCS-α expression. *SUCLA2-*shRNA treated neurons demonstrated a significant decrease in SCS A-β expression levels (**a**); while SCS G-β expression was not affected (**b**). The expression levels of SCS-α were decreased accompanying SCS A-β loss (**c**). The lower panels are representative immunoblots. TOM40 was used as the loading control. The data were collected from 3 independent experiments.

To determine the effect of SCS A-β deficiency on neurons, we exposed cultured mouse neurons to shRNA specifically targeting to *SUCLA2*. NonTarget shRNA was used as a critical control. The expression levels of SCS A-β were examined by immunoblotting. As expected, *SUCLA2* shRNA induced substantially decreased SCS A-β expression levels (Fig. 1a. P < 0.01). It would be of interest to know if the modulation of SCS A-β arouses compensatory changes of SCS G-β. To address this question, we further examined the expression levels of SCS G-β. There was only a marginal increase in the expression levels of SCS G-β by ~9% in SCS A-β down-regulated neurons (Fig. 1b. P > 0.05); while to our surprise we have found a remarkable reduction in the expression levels of SCS-α along with the loss of SCS A-β (Fig. 1c. P < 0.05). The unexpected down-regulation of SCS-α suggests that SCS A-β loss may induce overall reduction of SCS function in neurons.

### Loss of SCS A-β suppresses neuronal mitochondrial bioenergetics

**Figure 2:**
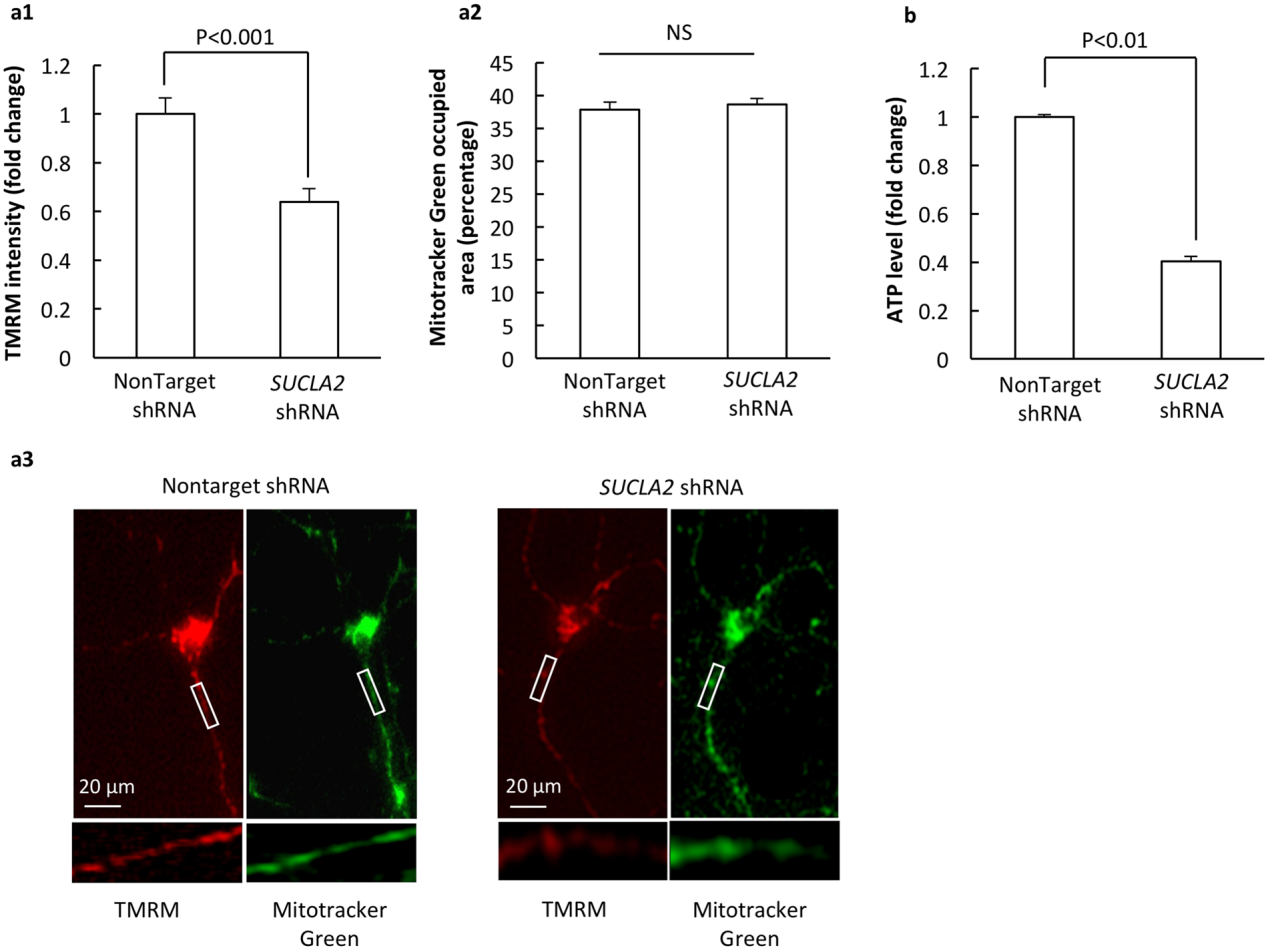
Loss of SCS A-β suppresses mitochondrial function. SCS A-β knockdown neurons showed decreased mitochondrial membrane potential (**a1**) and ATP content (**b**); while there was no significant difference in dendritic mitochondrial content between the two groups of neurons (**a2**). (**a3**) are representative images of TMRM (red) and Mitotracker Green (green) staining. The data were collected from 3 independent experiments. Scale bar = 20 µm.

Neurons predominantly rely on mitochondrial oxidative phosphorylation (OXPHOS) to produce ATP; while SCS plays an essential role in TCA cycle to generate substrates for mitochondrial respiration. To determine the effect of SCS A-β loss on neuronal OXPHOS efficiency we first examined mitochondrial membrane potential (mΔΨ), which is the result of mitochondrial respiration and the driving force for ATP generation via OXPHOS^21^. mΔΨ was determined by the staining of Tetramethylrhodamine, methyl ester (TMRM), a cell-permeant fluorescent indicator of mitochondrial membrane potential^19^. To exclude the possibility that altered TMRM intensity is due to changes in mitochondrial population, we double labeled neurons with Mitotracker Green to visualize neuronal mitochondria for the measurement of mitochondrial content. In comparison to nonTarget shRNA-treated neurons, dendritic mitochondria in SCS A-β down-regulated neurons exhibited a ~36% decrease in their TMRM intensity (Fig. 2a1 and 3. P < 0.001); while there was no significant difference in total dendritic mitochondrial content (Fig. 2a2 and 3). Therefore, the results indicate that SCS A-β loss induces neuronal mitochondrial membrane potential collapse, which is a demonstration of potentially impaired mitochondrial OXPHOS efficiency. ATP is the final product of mitochondrial OXPHOS^22^. We next measured ATP content levels by using the chemiluminescence method^19^. Consistent with the reduction of mΔΨ, *SUCLA2* shRNA-treated neurons exhibited a remarkable decrease in their ATP content levels as compared with their control counterparts (Fig. 2b. P < 0.01). Put together, the above results suggest that the suppression of SCS A-β expression dampens the oxidative metabolism of ATP production in neurons.

### Loss of SCS A-β promotes neuronal mitochondrial ROS generation

**Figure 3:**
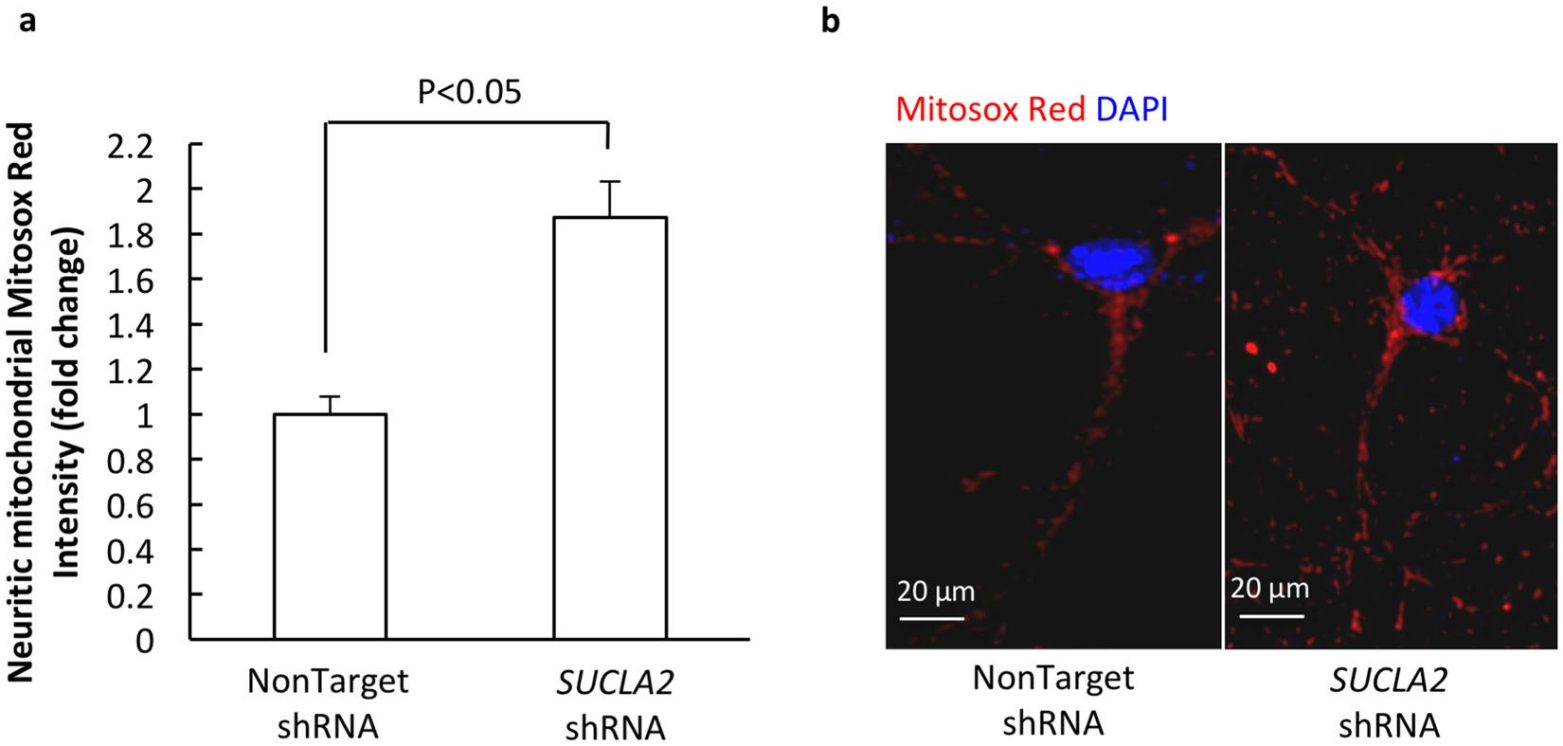
Loss of SCS A-β enhances neuronal mitochondrial superoxide production. *SUCLA2* shRNA-challenged neurons had significantly increased levels of Mitosox Red intensity (**a**). (**b**) are the representative images of Mitosox Red (red) staining. The nuclear was visualized by the staining of DAPI (blue). The data were collected from 3 independent experiments. Scale bar = 20 µm.

Loss of SCS A-β disrupts neuronal mitochondrial OXPHOS, which may potentiate ROS production. We then tested whether SCS A-β deficiency induces mitochondrial oxidative stress. NonTarget- and *SUCLA2* shRNA-treated neurons were incubated with MitoSox Red, which is a sensitive probe with its fluorescent intensity in proportionate to mitochondrial superoxide levels^23^. We have found that the down-regulation of SCS A-β induced a remarkable increase in MitoSox Red fluorescence (Fig. 3a and b. P < 0.05), indicating the close association of SCS deregulation with mitochondrial oxidative stress.

### Loss of SCS A-β leads to neuronal mtDNA depletion

**Figure 4:**
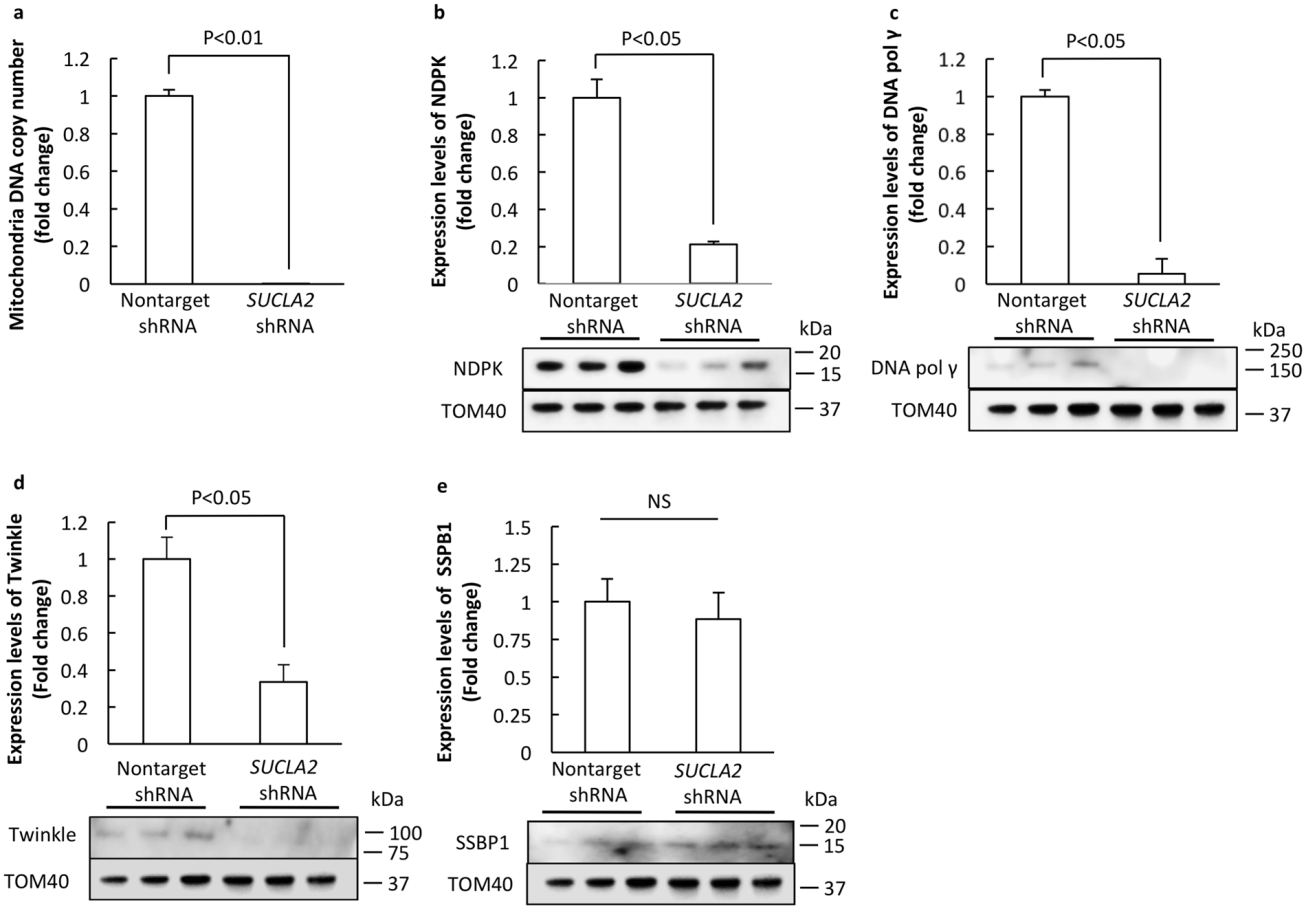
Loss of SCS A-β induces neuronal mtDNA depletion. (**a**) mtDNA copy number in *SUCLA2-* and nonTarget-shRNA-treated neurons. Nuclear DNA was used as the loading control. The expression levels of NDPK (**b**), DNA pol γ (**c**), Twinkle (**d**) and SSBP1 (**e**) in *SUCLA2-* and nonTarget-shRNA-treated neurons. The lower panels are representative immunoreactive bands. TOM40 was used as the loading control. The data were collected from 3 independent experiments.

Clinical observation of mtDNA depletion in mutant *SUCLA2* carriers indicates the role of SCS A-β in maintaining mtDNA stability^24^. To determine whether SCS A-β deficiency affects mtDNA in neurons we examined mtDNA abundance in nonTarget and *SUCLA2* shRNA-treated neurons by using real-time PCR. Nuclear DNA was used as the loading control. *SUCLA2* shRNA-treated neurons exhibited a significant decrease in their mtDNA copy number to a drastically low level (Fig. 4a. P < 0.01). Previous studies have attributed the effect of SCS A-β loss-of-function induced mtDNA depletion to the deregulation of NDPK^9^. We then examined the expression levels of NDPK in SCS A-β-deficient neurons. Our results showed a significant reduction of NDPK accompanying SCS A-β loss (Fig. 4b. P < 0.05). Furthermore, SCS A-β deficient neurons also demonstrated remarkably decreased expression levels of mitochondrial DNA pol γ (Fig. 4c. P < 0.05) and Twinkle (Fig. 4d. P < 0.05); while the levels of SSBP1 were relatively preserved (Fig. 4e). The above results have confirmed the detrimental influence of SCS A-β deregulation on neuronal mtDNA metabolism in neurons. The depletion of mtDNA induced by loss of SCS A-β may be a synergistic result of reduced NDPK and mitochondrial transcription factors including DNA pol γ and Twinkle.

### Loss of SCS A-β affects mitochondrial dynamics proteins

**Figure 5:**
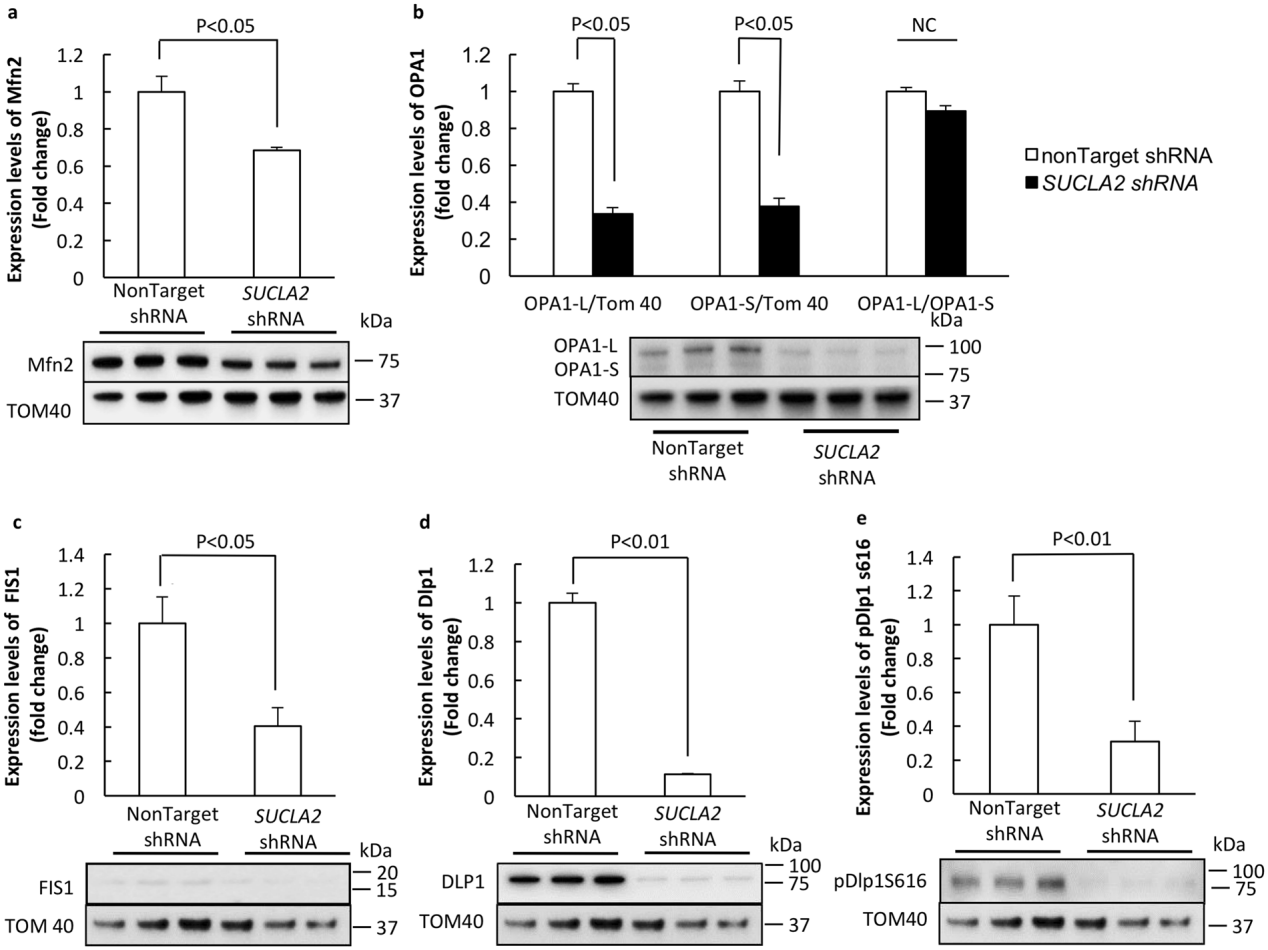
Loss of SCS A-β suppresses the expression of mitochondrial fusion and fission proteins. The expression levels of Mfn2 (**a**), OPA1 (**b**), FIS1 (**c**), Dlp1 (**d**) and pDlp1S616 (**e**) were measured by using immunoblotting. The lower panels are representative immunoblots. The data were collected from 3 independent experiments.

Mitochondrial dynamics is controlled by mitochondrial fusion and fission proteins^12^. To determine whether SCS A-β loss affects mitochondrial fusion and fission proteins we collected nonTarget shRNA- and *SUCLA2* shRNA-treated neurons for immunoblotting to examine the expression levels of mitochondrial fusion proteins including mitofusin 2 (Mfn2) and Optic atrophy 1 (Opa1), and mitochondrial fission proteins including mitochondrial fission 1 (Fis 1) and dynamin-like protein 1 (Dlp1) as well as phosphorylated Dlp1 (pDlp1 S616). As shown in Fig. 5a and b, SCS A-β deficiency induced significantly decreased expression levels of Mfn2, Opa1 long (Opa1-L) and short (Opa1-S) isoforms. But SCS A-β has little effect on the ratio of Opa1-L to Opa1-S (Fig. 5b). Interestingly, mitochondrial fission proteins were also suppressed by the loss of SCS A-β. SCS A-β down-regulated neurons underwent dramatically decreased levels of Fis1 (Fig. 5c. P < 0.05), Dlp1 (Fig. 5d. P < 0.01) and pDlp1 S616 (Fig. 5e. P < 0.01). The results implicate that loss of SCS A-β suppresses both mitochondrial fusion and fission proteins.

### Loss of SCS A-β induces neuritic mitochondrial morphological change

**Figure 6:**
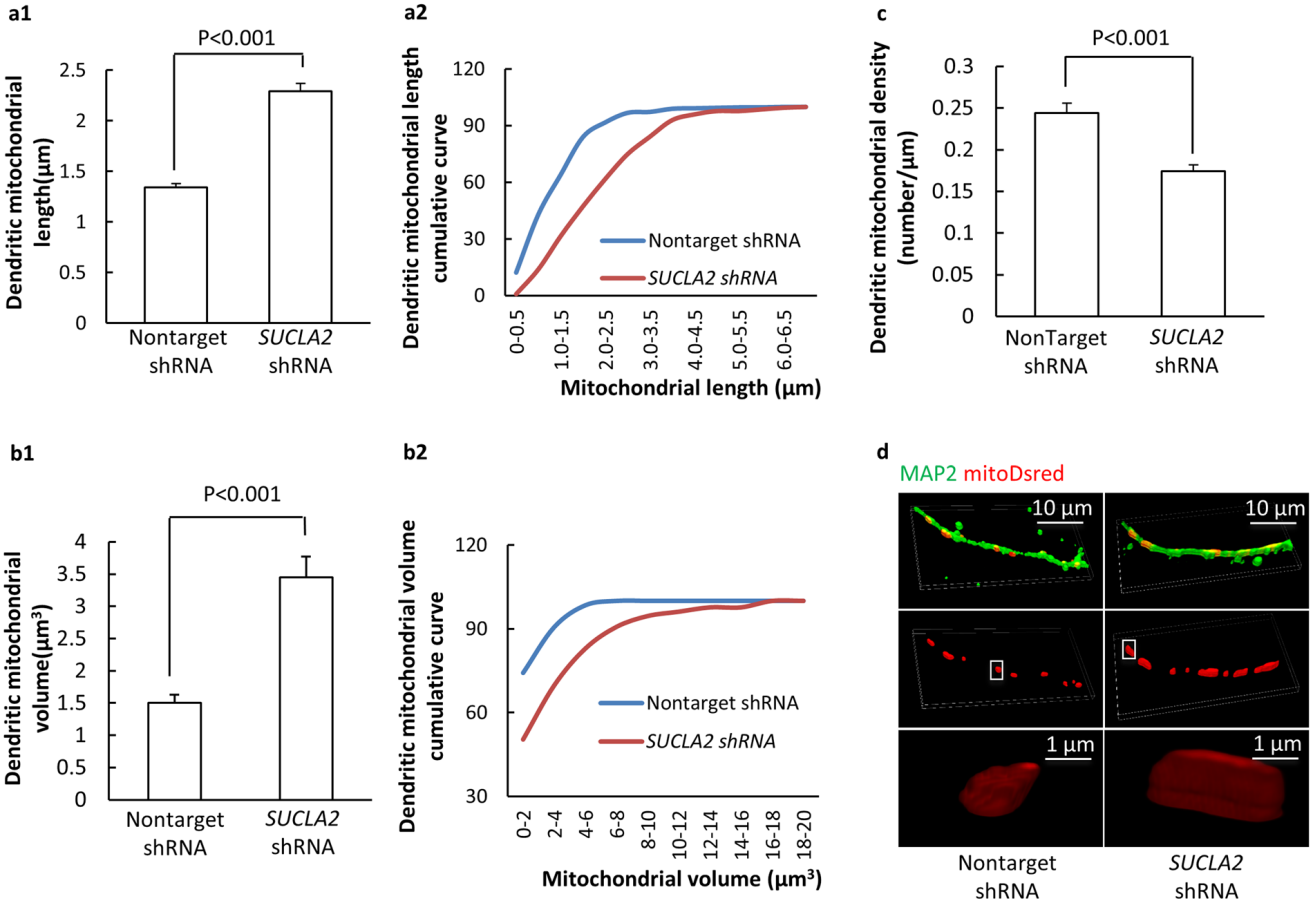
Loss of SCS A-β disrupts neuronal mitochondrial dynamics. (**a1**) Dendritic mitochondrial length in *SUCLA2-* and nonTarget-shRNA-treated neurons. (**a2**) Analysis of the cumulative fraction of mitochondrial length. Data were collected from 400 mitochondria. (**b1**) Dendritic mitochondrial volume in *SUCLA2-* and nonTarget-shRNA-treated neurons. (**b2**) Analysis of the cumulative fraction of mitochondrial volume. Data were collected from 400 mitochondria. (**c**) Dendritic mitochondrial density in *SUCLA2-* and nonTarget-shRNA-treated neurons. Data were collected from 24 neurons *per* group. (**d**) are r﻿epresentative images of mitoDsred. Scale bar = 10 µm for upper panels, = 1 µm for enlarged mitocho﻿ndria.

Changes in mitochondrial fusion and fission protein expression levels are strongly associated with altered mitochondrial dynamics. Therefore, we next examined the effect of SCS A-β loss on mitochondrial dynamics in neurons. Given SCS A-β down-regulation induces mitochondrial ROS over-production and the conventional fluorescent probe (Mitotracker Red) is ROS sensitive, we employed mitoDsred to label mitochondria^25^. The DNA of mitoDsred was delivered into neurons by lentivirus. Neuronal dendrites were recognized by the staining of MAP2. Analysis of the data showed that the average length of dendritic mitochondria was substantially increased by ~70% in the SCS A-β knock-down neurons in comparison to that in the nonTarget shRNA-exposed neurons (Fig. 6a1. P < 0.001). Moreover, the cumulative analysis of mitochondrial length in *SUCLA2* shRNA-treated neurons demonstrated a significant right shift towards increased mitochondrial length (Fig. 6a2). However, to determine whether mitochondria are enlarged in their size, the measure of the longitudinal change of mitochondrial size is not sufficient. In this regard, we further measured and compared mitochondrial volume. Similar to the changes in mitochondrial length, we have found that SCS A-β deficient neurons had a substantial increase in their dendritic mitochondrial volume as compared to their nonTarget shRNA-treated counterparts (Fig. 6b1. P < 0.001). The trend of increased mitochondrial volume with the loss of SCS A-β was further confirmed by a right shift of the cumulative curve of mitochondrial volume (Fig. 6b2). Furthermore, by analyzing mitochondrial density in dendrites we have observed a significant reduction of mitochondrial density in SCS A-β down-regulated neurons (Fig. 6c. P < 0.001). Put together, the results indicate that suppression of SCS A-β induces altered neuronal mitochondrial dynamics balance towards fusion.

### Loss of SCS A-β reduces synaptic density

**Figure 7:**
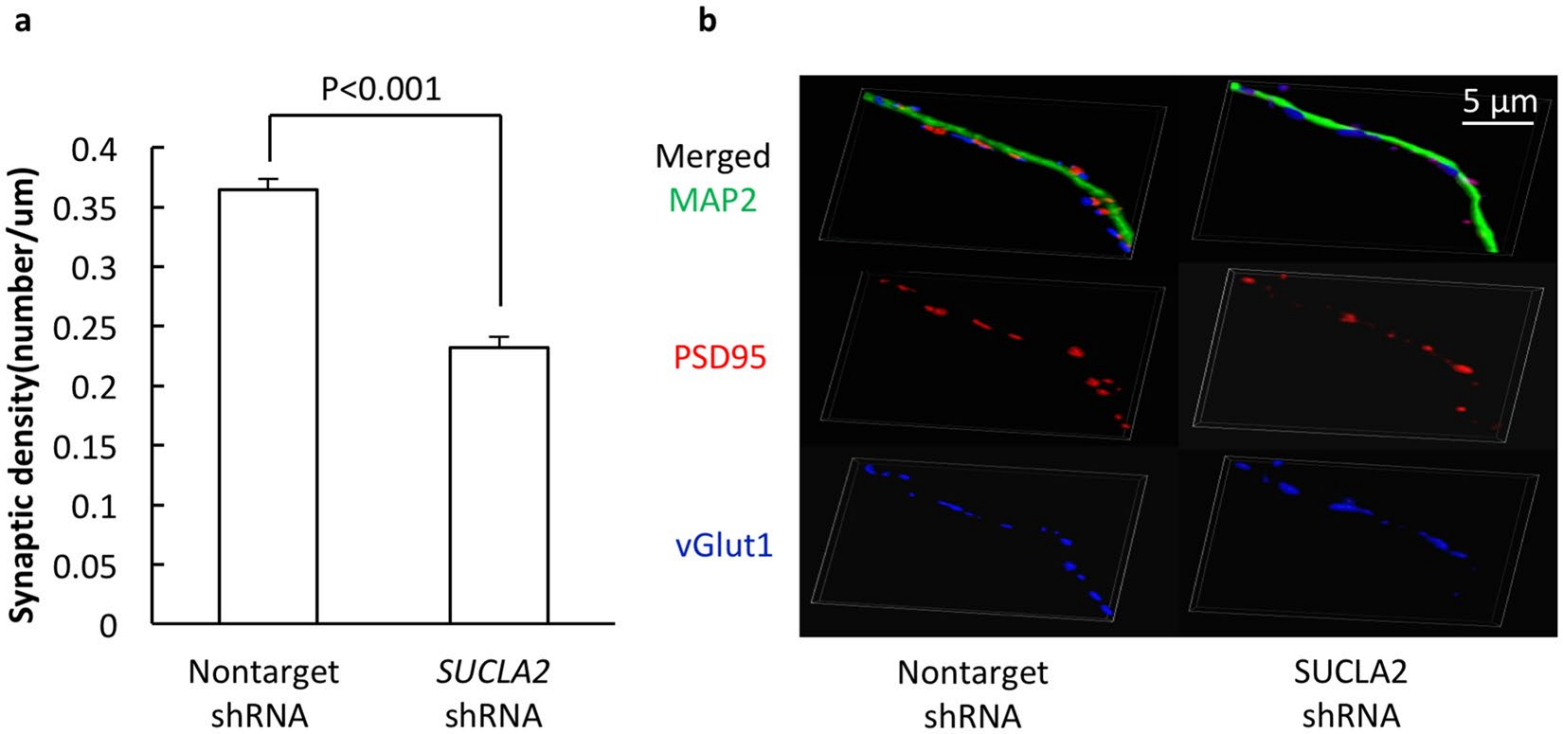
Loss of SCS A-β induces synaptic loss. (**a** and **b**) The co-staining of PSD95 (red) and vGlut1 (blue) Synapses were used to identify synapses. MAP2 (green) was used to determine the dendrites. *SUCLA2-*shRNA-treated neurons showed a significant decrease in their synaptic density. Data were collected from 27 neurons *per* group. Scale bar = 10 µm.

Synapses are the structural basis for inter-neuronal communication. Synaptic plasticity and synaptogenesis significantly rely on mitochondria to provide energy and maintain intra-synaptic calcium homeostasis^18^. The concurrence of mitochondrial dysfunction and synaptic failure has been repeatedly implicated at pathological states. In order to determine the detrimental impact of SCS A-β deficiency on synapses, we compared synaptic density between nonTarget and *SUCLA2* shRNA-challenged neurons. Synapses were identified by the staining of PSD95 (post-synaptic marker, Red color) and vGlut1 (pre-synaptic marker, Blue color). Dendrites were determined by the staining of MAP2 (Green color). Our results showed that loss of SCS A-β induced a significant reduction in synaptic density by ~37% (Fig. 7a and b. p < 0.001) as compared to that in the nonTarget shRNA-treated neurons. The results suggest that SCS A-β deficiency has deleterious effect on synapses, which is probably due to neuronal mitochondrial dysfunction.

## Discussion

SCS is an essential mitochondrial enzyme, which has multiple lines of critical functions. It catalyzes the reverse reaction of succinyl-Coenzyme A (succinyl-CoA) to CoA and succinate, thus driving the TCA cycle and the related mitochondrial metabolism^26^. In addition, emerging evidence has suggested the role of this enzyme in maintaining mitochondrial DNA (mtDNA) stability^4, 7^. In this study, we have found that loss of SCS A-β subsequently induces reduced expression of SCS-α in neurons; while SCS G-β is relatively unaffected. In our unpublished data, we have also seen the down-regulation of SCS-α in *SUCLA2* knock-out Hek-293 cells using CRISPR/Cas9 system. Given the similar result by using two different systems to induce SCS A-β loss in two different types of cells, we do not think that such change is the result of the off-target of gene manipulation. Therefore, it seems to suggest that the expression of SCS A-β and SCS-α is closely linked. Noteworthy, in a recently published study, Kacso and colleagues generated a heterogeneous mouse model with the deletion of one *SUCLA2* allele and found a significant decline in the expression levels of brain SCS-α subunit in these *SUCLA2* +/− mice^8^. Moreover, with age *SUCLA2* +/− mice exhibited no change (3 months old) to significantly increased levels (6 and 12 months old) of SCS G-β expression^8^. Such results are similar to our observations and serve as a piece of strong *in vivo* evidence showing the negative influence of SCS A-β loss on SCS-α expression. The reduction of SCS-α accompanying SCS A-β deficiency may be the cellular response to lowered SCS A-β levels; while it cannot be excluded that SCS A-β may regulate the expression of SCS-α through other yet-unknown mechanisms. But the concurrent reduction of SCS A-β and SCS-α indicates that SCS A-β loss causes an overall decrease of SCS activity. Previous clinical studies have shown that the deficiency of either SCS A-β or SCS-α induces similar symptoms, but SCS-α dysfunction has a more severe impact^7, 24^. Therefore, the changes that we have observed in this study may be the synergistic result of the SCS A-β and SCS-α deficiency.

In addition to suppressed neuronal mitochondrial metabolism and increased ROS production, we have also detected lessened mtDNA abundance in SCS A-β-deficient neurons. Such change correlates to previous clinical observations in patients carrying mutations in *SUCLA2* (encoding SCS A-β) and *SUCLG1* (encoding SCS-α)^10^ as well as recent findings in *SUCLA2* +/− mice^8^. Here, we have found that in addition to the reduction in NDPK levels, two critical proteins related to mtDNA replication including Twinkle and mitochondrial DNA pol γ also decreased as the result of SCS A-β knock-down. We think that the combination of the changes of NDPK, Twinkle and mitochondrial DNA pol γ together underlie mtDNA depletion in SCS A-β-deficient neurons, which has verified the key role of SCS in maintaining mtDNA stability. Notably, Emerging evidence has suggested the association of deficits in Mfn2 and Opa1 with mtDNA instability^27^; while we have found that loss of SCS A-β causes substantially decreased Mfn2 and Opa1 expression levels. In this regard, mitochondrial fusion and fission deregulation is as well potentially involved in SCS A-β deficiency-mediated mtDNA depletion. However, a previous study using fibroblast cells from a SCS A-β -deficient patient found no change in mtDNA levels; while the down-regulation of SCS G-β in SCS A-β -deficient fibroblast cells caused a prominent mtDNA loss^9^. The authors proposed that it is a manifestation of tissue-specific vulnerability to SCS A-β deficiency^9^. Indeed, it has been suggested that the abundance of the two SCS β isoforms is variable in different types of tissues. Generally saying, SCS G-β is highly expressed in tissues with more active biosynthesis i.e. the liver; while SCS A-β has higher expression levels in tissues with intensive oxidative metabolism such as the brain and skeletal muscles^2–4^. In this regard, this tissue-specific expression pattern seems to suggest that SCS A-β is important for neuronal mtDNA metabolism, which is supported by our results.

An interesting finding of this study is the imbalanced mitochondrial dynamics towards fusion with dramatic reduction in the expression levels of both fusion and fission proteins, which has raised an intriguing scientific question. This is because decreased expression levels of mitochondrial fission proteins including Fis1, Dlp1 and phosphorylated Dlp1 may promote mitochondrial fusion^28^. However, the increased mitochondrial size does not comply with decreased expression levels of Mfn2 and Opal. A possible reason is that the down-regulation of mitochondrial fusion proteins in SCS A-β deficient neurons is a secondary response to compensate the deregulation of fission proteins. In addition, Opa 1 is a GTPase whose function relies on the availability of GTP. SCS is the primary source of GTP in mitochondria. It could not be excluded that preserved SCS G-β in SCS A-β deficient neurons shifts the ratio of SCS ATP/GTP production towards GTP generation. In this regard, mitochondrial fusion and fission proteins are rebalanced at lower expression levels; while the relative abundance of mitochondrial GTP favors the function of Opa1^2^. But this postulation could arise an argument given that Dlp1 is as well a GTPase^29^. To address this question, it should be noted that Dlp1 is a cytosolic protein and does not locate in mitochondrial matrix^29^. Therefore, altered intra-mitochondrial GTP production caused by SCS deactivation may have little or no effect on Dlp1 function. Another question that merits discussion is that it is generally accepted that mitochondrial fragmentation is a sign of mitochondrial dysfunction at pathological states; while mitochondrial fusion is beneficial. Here, we have found that SCS A-β deficiency results in mitochondrial dysfunction with enlarged mitochondria. This seems to add credibility to the hypothesis that the balance of mitochondrial dynamics but not fission or fusion *per se* is critical for mitochondrial function, which has been suggested previously^30^. However, the answers to these questions need our further investigation.

Lastly, neurons are polar cells with long processes stemming from the soma; while mitochondria are scarcely distributed in dendrites and axons. Synaptic activity predominantly relies on neuritic mitochondrial function^18^. Therefore, it is not surprising that we have observed substantially decreased synaptic density along with SCS A-β loss-induced neuronal mitochondrial dysfunction and dynamics deregulation.

In summary, we have shown in this study that SCS A-β loss mediates severe mitochondrial dysfunction including compromised OXPHOS efficiency, increased ROS generation, mtDNA depletion as well as altered mitochondrial dynamics towards fusion. These mitochondrial defects have detrimental effects on neurons, causing the decline of synaptic density. The results have furnished our understanding of the pathological influence of SCS A-β deficiency and formed the groundwork for our future study on the physiological role of SCS in neurons.

### Data availability statement

The datasets generated or analyzed during the current study are available from the corresponding author on reasonable request.
